# Proteomic Analysis of the Hemolymph After *Metschnikowia bicuspidata* Infection in the Chinese Mitten Crab *Eriocheir sinensis*

**DOI:** 10.3389/fimmu.2021.659723

**Published:** 2021-04-01

**Authors:** Hongbo Jiang, Jie Bao, Yuenan Xing, Chengcheng Feng, Xiaodong Li, Qijun Chen

**Affiliations:** Key Laboratory of Livestock Infectious Diseases in Northeast China, Ministry of Education, Key Laboratory of Zoonosis, Shenyang Agricultural University, Shenyang, China

**Keywords:** *Metschnikowia bicuspidata*, *Eriocheir sinensis*, milky disease, proteome, immune response

## Abstract

The “milky disease” of the Chinese mitten crab, *Eriocheir sinensis*, is a highly lethal fungal disease caused by *Metschnikowia bicuspidata* infection. To elucidate the immune responses of the hemolymph of *E. sinensis* to *M. bicuspidata* infection, a comparative analysis of the hemolymph of *E. sinensis* infected with *M. bicuspidata* and that treated with phosphate buffered saline was performed using label-free quantitative proteomics. A total of 429 proteins were identified. Using a 1.5-fold change in expression as a physiologically significant benchmark, 62 differentially expressed proteins were identified, of which 38 were significantly upregulated and 24 were significantly downregulated. The upregulated proteins mainly included cytoskeleton-related proteins (myosin regulatory light chain 2, myosin light chain alkali, tubulin α-2 chain, and tubulin β-1 chain), serine protease and serine protease inhibitor (clip domain-containing serine protease, leukocyte elastase inhibitor, serine protein inhibitor 42Dd), catalase, transferrin, and heat shock protein 70. Upregulation of these proteins indicated that phenoloxidase system, phagocytosis and the ROS systems were induced by *M. bicuspidata*. The downregulated proteins were mainly organ and tissue regeneration proteins (PDGF/VEGF-related factor protein, integrin-linked protein kinase homing pat-4 gene) and hemagglutination-associated proteins (hemolymph clottable protein, hemocyte protein-glutamine gamma-glutamyltransferase). Downregulation of these proteins indicated that *M. bicuspidata* inhibited hemocyte regeneration and hemolymph agglutination. Fifteen differentially expressed proteins related to immunity were verified using a parallel reaction monitoring method. The expression trend of these proteins was similar to that of the proteome. To the best of our knowledge, this is the first report on the proteome of *E. sinensis* in response to *M. bicuspidata* infection. These results not only provide new and important information on the immune response of crustaceans to yeast infection but also provide a basis for further understanding the molecular mechanism of complex host pathogen interactions between crustaceans and fungi.

## Introduction

The Chinese mitten crab, *Eriocheir sinensis*, is a high-value economic aquatic product that is widely distributed in China’s lakes and reservoirs. Fishery statistics from 2019 showed that the breeding output of *E. sinensis* reached 778,682 tons in China (1) and *E. sinensis* aquaculture has become an important pillar industry. With the rapid development of intensive culture of *E. sinensis*, various viral and bacterial diseases are causing serious damage to the aquaculture industry, leading to significant economic losses. At present, the reported diseases include *E. sinensis* tremor disease caused by *Spiroplasma eriocheiris* infection (2), vibrio disease caused by *Vibrio parahaemolyticus* and *V. anguillarum* (3, 4), hepatopancreatic necrosis disease caused by microsporidia (5), and parasitic diseases caused by ciliates (6). In 2018, some diseased *E. sinensis* with a milky-like liquid accumulated *in vivo* were found in Panjin city in the Liaoning province, and this condition was referred to as “milky disease.” After infection, the activity of infected crabs was weakened, their pereiopods easily fell off, and the crabs died. The infection rate was greater than 20% (7). The pathogen was identified as *Metschnikowia bicuspidata*. The incidence of infection has now increased and the pathogen has spread to many cities and provinces in China. *M. bicuspidata* can infect not only invertebrates such as Artemia, Daphnia, *Portunus trituberculatus*, and *Macrobrachium rosenbergii*, but also fish (8–11). Similar to *P. trituberculatus* and *M. rosenbergii*, infected *E. sinensis* also showed milky symptoms. To the best of our knowledge, there are no effective prevention and control measures for “milky disease”, although some progress has been made in *in vitro* experiments (12, 13).

It is well known that vertebrates have both innate and acquired immune systems; however, crustaceans only rely on innate immune systems to defend against foreign pathogens. As an important part of crustacean immunity, hemolymph plays a critical role in the process of crustacean resistance to foreign pathogens through phagocytosis, covering, and wound repair functions (14). Therefore, using hemolymph as the research object can better clarify the immune mechanism. In immunological research, transcriptomics and proteomics have been widely used to study the interaction between crustaceans and pathogens (15–17). Proteomics was able to identify the proteins and pathways involved in immune responses more accurately than transcriptomics, by analyzing the changes in host protein expression before and after pathogen invasion. In crustaceans, numerous immune-related proteins and pathways involved in pathogen infection were identified by describing their proteomic characteristics (18, 19). For example, the interaction between red claw crayfish and white spot syndrome virus (WSSV) *in vitro* was studied using proteomics (20). Twenty differential proteins in hemolymph that were involved in the immune responses of *E. sinensis* to *S. eriocheiris* infection were obtained using proteomics techniques (21). Sun et al. (22) used proteomics techniques to study the immune response of mud crabs to WSSV or *V. alginolyticus* infection. Therefore, proteomics is an excellent method for studying the immune response of crustaceans to yeast fungal infection.

The purpose of this study was to identify changes in protein expression in the hemolymph and elucidate the immune response of *E. sinensis* at the translational level after being challenged with *M. bicuspidata*. These results will not only provide new and important information on the immune response of crustaceans to yeast infection but will also provide a basis for further understanding the molecular mechanism of complex host pathogen interactions between crustaceans and fungi.

## Materials and Methods

### Pathogen Challenge

*Eriocheir sinensis* (20 ± 3 g, 1: 1 female: male ratio) were purchased from a market in Panjin city, China, transported back to Shenyang Agricultural University, and temporarily cultured in 300 L tanks. Healthy *E. sinensis* [verified by *M. bicuspidata* negative results using PCR D1/D2 domain of the 26S rDNA sequence analyses (7)] were cultured for 7 d prior to the experimental tests. The control group (n = 30) was injected with 100 μL of phosphate-buffered saline (pH 7.4), and the experimental group (n = 30) was injected with 100 μL of *M. bicuspidata* (10^7^ cells/mL). At 48 h post-infection, 15 individuals from each group were randomly selected, and the hemolymph was extracted from the last pereiopod with a 1 mL syringe and mixed with an equal volume of sterile anticoagulant citrate glucose solution B (21). Three biological replicates with five organisms mixed per replicate were performed in the two groups, amounting to six samples. The samples were frozen in liquid nitrogen and refrigerated at −80°C for further proteomic studies.

### Protein Extraction

After sample refrigeration, 1% protease inhibitor was added to each group. The samples were then centrifuged at 4°C and 12000 × *g* for 10 min to remove cell debris. The supernatant was extracted and its protein concentration was determined using a BCA kit (Nanjing Jiancheng Bioengineering Institute, Nanjing, China), following the manufacturer’s instructions.

### Trypsin Digestion

Fifty micrograms of extracted proteins were enzymolyzed and the volume was adjusted to 200 μL with lysis buffer (8 M urea, 1% Triton 100, 10 mM dithiothreitol, and 1% protease inhibitor cocktail). An equal volume of precooled acetone was added. After vortex mixing, four times the volume of precooled acetone was added and precipitated at −20°C for 2 h. The supernatant was discarded after centrifugation at 4500 × *g* for 5 min. The precipitate was washed twice with precooled acetone and air-dried. After adding 200 mM tetraethylammonium bromide, the precipitate was dispersed using ultrasound. Next, 1:50 (trypsin: protein, M/M) trypsin was added for overnight enzymolysis. Subsequently, dithiothreitol was added (5 mM final concentration), and the sample was reduced for 30 min at 56°C. The final concentration of iodoacetamide was 11 mM, and this was incubated in the dark at 20°C for 15 min.

### Analysis *via* Liquid Chromatography-Mass Spectrometry (LC-MS)/MS

The peptides were dissolved in mobile phase A and separated using an EASY-nLC 1000 ultra-performance liquid chromatography system (Thermo Fisher Scientific, Waltham, MA, USA). Both mobile phase A and B were aqueous solutions containing 0.1% formic acid and 2% acetonitrile, and 0.1% formic acid and 90% acetonitrile, respectively. The flow rate of the liquid was 450.00 nL/min, gradient settings were 0–90 min, 6–24% B; 90–114 min, 24–35% B; 114–117 min, 35–80% B; 117–120 min, 80% B. After ionization (nanoelectrospray ionization source, electrospray voltage: 2.1 kV), the peptide was analyzed using Q Exactive™ Plus (Thermo Fisher Scientific) mass spectrometry, and the parent ion and its secondary fragments were detected and analyzed using high-resolution Orbitrap. The scanning ranges and resolutions were respectively 350–1800 m/z and 70000.00 for the primary MS, and 100 m/z and 17500.00 for the secondary MS. A data-dependent scanning program was used during the data acquisition mode. The automatic gain control was set to 5E4, the signal threshold was set to 4E4 ions/s, and the maximum injection time was set to 50 ms.

### Database Search

The secondary MS data were retrieved using Maxquant 1.5.2.8. Retrieval parameter settings were as follows: the database was *E. sinensis* (6843 sequences); to eliminate the influence of contaminated proteins in the identification results, a common contaminated library was added to the database. The cleavage enzyme was set as Trypsin/P; the number of missing sites was set as two. The minimum length of the peptide was set as seven amino acid residues; the maximum modification number of the peptide was set as 5. The mass error tolerance of primary parent ion was 10.0 ppm and 5 ppm for the first and main searches, respectively. The mass error tolerance of the secondary fragment ion was 0.02 Da. Carbamidomethyl on Cys was specified as fixed and acetylation modifications, and oxidation on Met and deamidation on Asn and Gln were specified as variable modifications. The quantitative method was set to label-free quantification, and the false positive rate of protein identification and peptide spectrum match identification was set to 1%.

### Protein Annotation and Function Enrichment

#### Gene Ontology (GO) Analysis

First, the system converted the protein ID to a UniProt ID, then used the UniProt ID to match a GO ID, and extracted the corresponding information from the UniProt-GOA database based on the GO ID. If there was no protein information in the UniProt GOA database, interproscan (http://www.ebi.ac.uk/interpro/), an algorithm software based on protein sequence, was used to predict the GO function of the protein. The proteins were then classified according to cell composition, molecular function, or physiological process.

#### GO and Pathway Enrichment Analyses

Fisher’s exact test was used to test differentially expressed proteins in the background of identified proteins. A p-value of less than 0.05 for the GO and pathway enrichment tests was considered significant. Finally, these channels were classified according to the KEGG channel level classification method.

#### Clustering Analysis Based on Functional Protein

First, we collected information on the functional classification and corresponding p-values of the protein groups, and then we screened out the functional classifications with significant enrichment (*p*-value < 0.05) in at least one protein group. The filtered *p*-value data matrix was first transformed by the logarithm of −log10, and then the transformed data matrix was classified using Z transformation. Finally, the hierarchical clustering (Euclidean distance, average connection clustering) method was used for unilateral clustering analysis of the data set obtained by Z-transform. The clustering relationship was visualized using the Heatmap 2 function in the R language package “gplots.”

### Targeted Protein Quantification *Via* Parallel Reaction Monitoring (PRM)

Fifteen differentially expressed proteins were randomly selected from different proteins for PRM verification. The peptide was derived from the remaining peptide of the proteome. The mobile phase composition, electrospray voltage, Orbitrap resolution, and mass spectrometry were consistent with the previously described methods The differences were as follows: the liquid gradient settings were 0–40 min, 6–25% B; 40–52 min, 25–35% B; 52–56 min, 35–80% B; 56–60 min, 80% B, with the flow rate maintained at 500 nL/min. Automatic gain control was set at 3E6 for full MS and 1E5 for MS/MS. The maximum injection time was set to 50 ms for full MS and 160 ms for MS/MS. For the target peptides, relative quantitative analysis was repeated three times after normalizing the quantitative information. Peptide parameters were as follows: protease was set to trypsin [KR/P] and the maximum number of missed cut sites and peptide length was set to 0 and 7–25 amino acid residues, respectively. Cysteine alkylation was set as fixed modification.

## Results

### Proteomics Overview

**Figure 1:**
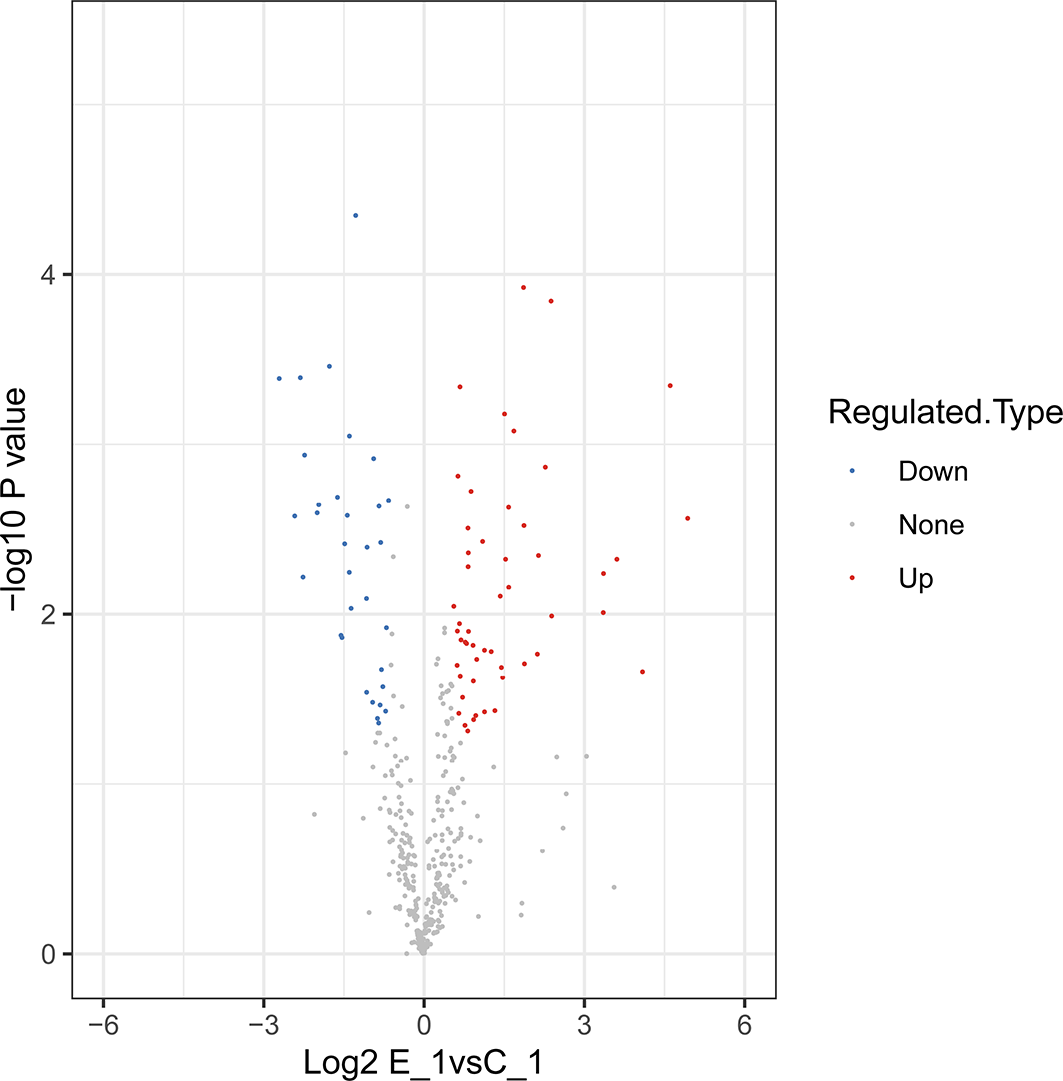
Volcano plots of differentially-expressed proteins.

**Figure 2:**
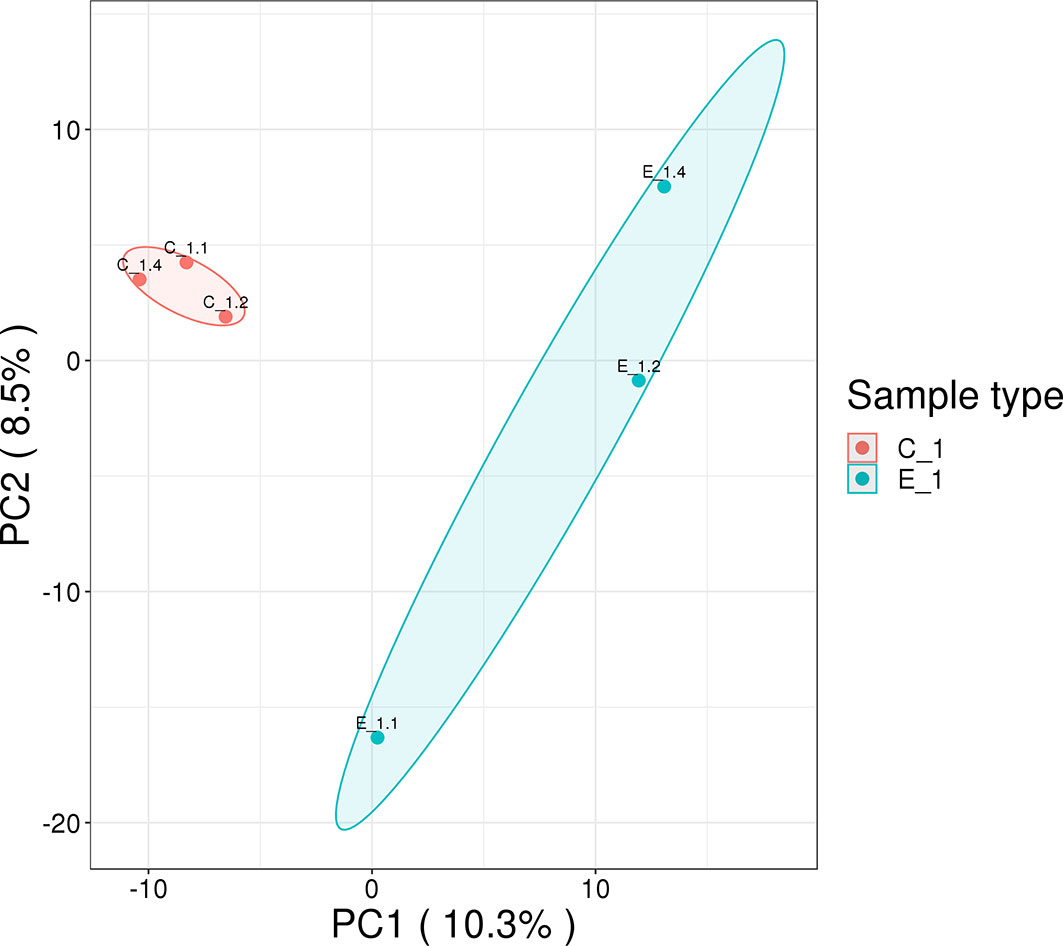
Principal component analysis of proteomics data. The distance of each sample point represents the degree of similarity between samples. The closer the sample points, the higher the similarity between samples.

In this study, 230,809 secondary spectrums were received by mass spectrometry. After searching the protein database, the number of available secondary mass spectra was 15,982 and the utilization rate was 6.9%. A total of 2892 peptides were identified *via* spectroscopic analysis, of which 2858 were found to be specific. A total of 429 proteins were identified (**Table S1**), of which 304 were quantifiable. Among the identified proteins, 62 were identified as DEPs, which used thresholds of a 1.5-fold (*p* < 0.05) increase or a 0.67-fold decrease (**Table S2**); of these, 38 were significantly upregulated and 24 were significantly downregulated after *M. bicuspidata* challenge (**Figure 1**). Principal component analysis results are shown in **Figure 2**. The immune-related proteins in these DEPs were shown in **Table 1**.

**Table 1:** Differentially expressed immune-related proteins.

Accession no.	Protein name	Fold change	*P*-value	Regulation Type
TRINITY_DN10_c0_g3_m.452	Cystatin-A	1.8689	0.041024621	Up
TRINITY_DN1001_c0_g1_m.15	Gelsolin, cytoplasmic	2.0479	0.002688917	Up
TRINITY_DN1355_c0_g1_m.1522	Purine nucleoside phosphorylase	1.9057	0.001507337	Up
TRINITY_DN1382_c1_g3_m.1620	Myosin regulatory light chain 2	2.6698	0.036741424	Up
TRINITY_DN1537_c1_g1_m.2248	Low-density lipoprotein receptor	3.2765	0.005054685	Up
TRINITY_DN182_c0_g1_m.3315	Myosin light chain alkali	2.1011	0.008237636	Up
TRINITY_DN187_c0_g1_m.3442	Tubulin alpha-2 chain	2.1529	0.000265648	Up
TRINITY_DN215_c0_g1_m.4297	Tubulin beta-1 chain	1.5009	0.045635628	Up
TRINITY_DN21871_c0_g1_m.4382	Heat shock cognate 70 kDa protein	1.7352	0.011368734	Up
TRINITY_DN2200_c0_g1_m.4428	L-ascorbate oxidase	5.7885	0.007828275	Up
TRINITY_DN2465_c1_g1_m.5252	Prosaposin	1.5803	0.018755506	Up
TRINITY_DN2539_c0_g1_m.5462	Transferrin	3.0141	0.016913764	Up
TRINITY_DN2787_c0_g1_m.6005	C-type lectin lectoxin-Enh6	1.9349	0.029630599	Up
TRINITY_DN284_c3_g3_m.6143	Serine protease inhibitor 42Dd	4.1181	0.049267135	Up
TRINITY_DN3715_c0_g1_m.7754	Arginine kinase	2.1531	0.009807981	Up
TRINITY_DN3897_c0_g1_m.7995	Tubulin alpha-1	1.6799	0.000044299	Up
TRINITY_DN418_c1_g1_m.8384	CLIP domain-containing serine protease 2	2.4207	0.011347414	Up
TRINITY_DN600_c0_g1_m.10539	protein spätzle 5-like isoform X2	3.3422	0.009098329	Up
TRINITY_DN799_c0_g1_m.12110	Leukocyte elastase inhibitor	4.9718	0.006077766	Up
TRINITY_DN8507_c0_g2_m.12453	Tubulin alpha-2/alpha-4 chain	1.7535	0.045897205	Up
TRINITY_DN925_c0_g2_m.12943	Catalase	2.4116	0.01111043	Up
TRINITY_DN941_c0_g1_m.13028	Hemolymph clottable protein	0.2775	0.003626456	Down
TRINITY_DN10547_c0_g1_m.243	Anti-lipopolysaccharide factor	0.5259	0.012217912	Down
TRINITY_DN106_c1_g2_m.316	Annexin A11	0.517	0.005016753	Down
TRINITY_DN13_c0_g1_m.1679	Alpha-N-acetylgalactosamine-specific lectin	0.3086	0.012753786	Down
TRINITY_DN16_c0_g2_m.2902	PDGF/VEGF-related factor	0.392	0.00340923	Down
TRINITY_DN1708_c2_g1_m.2937	Putative uncharacterized oxidoreductase C513.07	0.6154	0.00158104	Down
TRINITY_DN195_c4_g3_m.3711	Integrin-linked protein kinase homolog pat-4	0.6222	0.003803178	Down
TRINITY_DN559_c0_g3_m.9998	Hemocyte protein-glutamine gamma-glutamyltransferase	0.6299	0.029494743	Down

### GO and KEGG Analysis of DEPs

**Figure 3:**
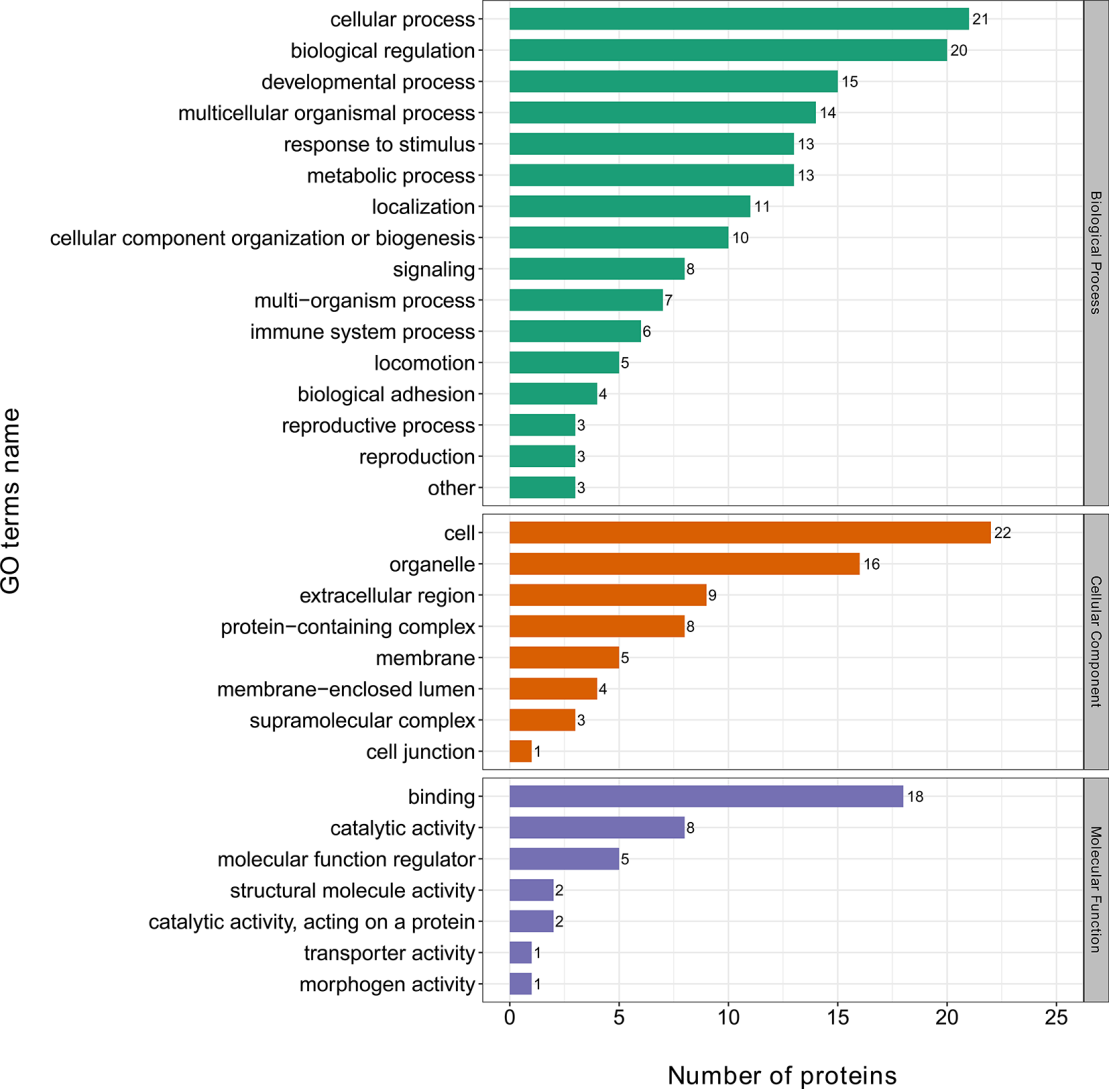
Gene ontology (GO) analysis of differentially expressed proteins.

The GO classification analysis of the DEPs showed that biological processes were mainly concentrated in cellular processes, biological regulation, the developmental process, the multicellular organismal process, response to stimulus, and the metabolic process. Cellular components were mainly concentrated in cells, organelles, extracellular regions, protein-containing complexes, and membranes; molecular functions were mainly enriched in binding, catalytic activity, molecular function regulation, and structural molecule activity (**Figure 3**).

**Figure 4:**
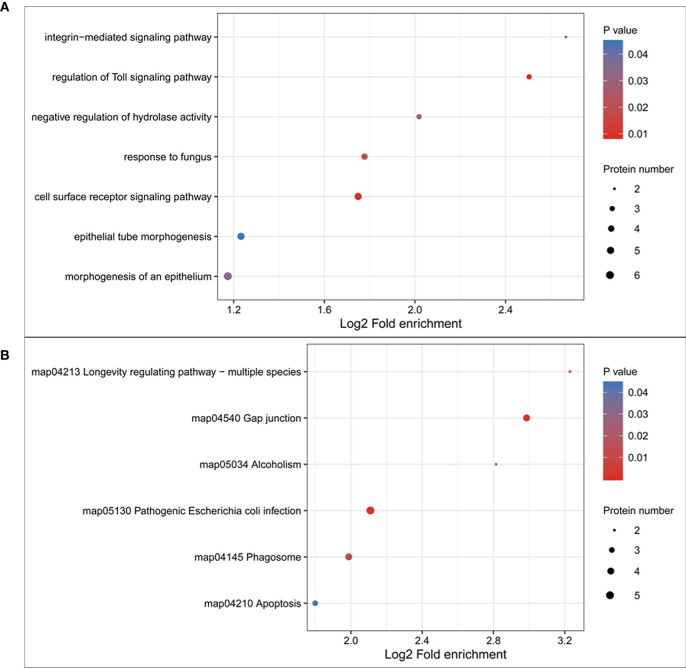
Gene ontology (GO) and KEGG pathway enrichment of the differentially expressed proteins. **(A)** GO pathway enrichment of differentially expressed proteins; **(B)** KEGG pathway enrichment of upregulated proteins.

Pathway analysis of the DEPs was conducted using GO and KEGG analyses. The results of biological processes pathway by GO analysis showed that the significant enrichment pathways included the integrin-mediated signaling pathway, regulation of toll signaling pathway, negative regulation of hydrolase activity, response to fungus, cell surface receptor signaling pathway, epithelial tube morphogenesis, and morphogenesis of an epithelium (**Figure 4A**). The results of the KEGG pathway analysis showed that the significant enrichment pathways for the upregulated proteins included those for longevity regulating pathway, gap junction, alcoholism, pathogenic *Escherichia coli* infection, phagosome, and apoptosis (**Figure 4B**). There was no significant enrichment pathway for the downregulated proteins.

### Validation of Proteome Data for Selected Proteins *via* PRM

In this research, we used the PRM method to verify the different expression proteins obtained from the proteome. Fifteen immune-related proteins from the DEPs were randomly selected for PRM analysis. Although the values of PRM and proteome were slightly different, the overall trend was highly consistent (**Table 2**), which showed that the results of our proteome study are reliable.

**Table 2:** Relative expression levels of selected proteins measured *via* parallel reaction monitoring (PRM).

Accession no.	Protein Name	Folder change of PRM	*P*-value	Folder change of proteome
TRINITY_DN21871_c0_g1_m.4382	Heat shock cognate 70 kDa protein	1.40	0.0165	1.74
TRINITY_DN2787_c0_g1_m.6005	C-type lectin lectoxin-Enh6	1.84	0.00414	1.93
TRINITY_DN1001_c0_g1_m.15	Gelsolin	1.87	0.000640	2.05
TRINITY_DN106_c1_g2_m.316	Annexin A11	0.30	0.00116	0.52
TRINITY_DN1537_c1_g1_m.2248	Low-density lipoprotein receptor	2.79	0.00429	3.28
TRINITY_DN1708_c2_g1_m.2937	Putative uncharacterized oxidoreductase	0.51	0.00217	0.62
TRINITY_DN215_c0_g1_m.4297	Tubulin beta-1 chain	1.37	0.0188	1.50
TRINITY_DN2200_c0_g1_m.4428	L-ascorbate oxidase	5.99	0.000318	5.79
TRINITY_DN2539_c0_g1_m.5462	Transferrin	2.27	0.0219	3.01
TRINITY_DN559_c0_g3_m.9998	Hemocyte protein-glutamine gamma-glutamyltransferase	0.58	0.0110	0.63
TRINITY_DN600_c0_g1_m.10539	Protein spaetzle 5-like isoform X2	2.71	0.00957	3.34
TRINITY_DN925_c0_g2_m.12943	Catalase	2.15	0.0357	2.41
TRINITY_DN10547_c0_g1_m.243	Anti-lipopolysaccharide factor	0.42	0.00140	0.53
TRINITY_DN418_c1_g1_m.8384	CLIP domain-containing serine protease 2	1.75	0.0407	2.42
TRINITY_DN941_c0_g1_m.13028	Hemolymph clottable protein	0.25	0.00205	0.28

## Discussion

With analytical technology development, proteomic research has gradually become an innovative field in the search for functional proteins. In this field, the differentially expressed proteins identified between the control and experimental groups can be easily explained to a high reasonable extent (23, 24). In some cases, such research can help us understand the response of cells to various external factors.

To the best of our knowledge, this is the first study on the proteomic characteristics of the hemolymph of *E. sinensis* in response to *M. bicuspidata*, using proteomic methods. Our results showed that the proteins and biological processes in the hemolymph changed significantly when *E. sinensis* was infected with *M. bicuspidata*. A total of 304 proteins were quantifiable, of which 62 were differentially expressed. We validated 15 differentially expressed proteins using MS-based precise quantitative PRM analysis. Similar to western blotting, PRM is a useful methodology for proteomics validation (25, 26). In the absence of specific antibodies, the detection time can be greatly shortened, and high accuracy can be maintained (27). In this study, the trend of the PRM was consistent with that of proteomics. Therefore, the results of mass spectrometry experiments are technically credible.

Based on the obtained proteins, the differentially expressed proteins may directly or indirectly participate in the immune response of *E. sinensis*. The KEGG enrichment results showed that cytoskeletal proteins, proteins with gap junctions, pathogenic *E. coli* infection, and the phagosome pathway in the hemolymph were influenced remarkably by *M. bicuspidata* infection. Cytoskeletal proteins, including myosin regulatory light chain (MRLC), myosin light chain (MLC), and tubulin α and β chains, were found to be significantly upregulated after *M. bicuspidata* infection. It is well known that cytoskeletal proteins are essential for phagocytosis, which is an important innate immune response of crustaceans (28, 29). The basic structure of myosin in vertebrate and invertebrate muscles is identical. A complete conventional myosin molecule is a hexamer composed of two myosin heavy chains, two MLC, and two MRLC. Myosin is a vital component of muscle cells and plays a crucial role in muscle movement, material transport, cytoplasmic flow, energy supply, and signal transduction (30). Han et al. (31) found that the expression of MLC gene significantly increased in WSSV-resistant *Marsupenaeus japonicus*. RNAi experiments showed that the phagocytic rate and phagocytic index significantly decreased after silencing of the MLC gene. Similarly, MRLC phosphorylation increases after YHV infection in *Penaeus monodon*, and inhibition of phosphorylation leads to increased YHV replication (32). These results suggest that MLC and MRLC might play an important role in crustacean defense against pathogen infection by regulating hemocyte phagocytic activity. In this study, MRLC and MLC protein expression levels significantly decreased in the hemolymph after stimulation with *M. bicuspidata*. Tubulin is a type of globulin, which is a heterodimer formed by the polymerization of α- and β-tubulin molecules. Each of these dimers is combined with two nucleotide molecules, one of which binds tightly, whereas the other binds loosely, and both can be exchanged rapidly. Tubulin is one of the main components of the cytoskeleton and plays an indispensable role in many processes, including structural support, intracellular transport, and DNA separation (33). In this study, tubulin α and β protein levels were significantly higher in the infection group than in the control group. Li et al. (34) found that after WSSV infection, tubulin α-1 and β-1 chains were significantly upregulated. Meng et al. (21) also found that tubulin α gene expression in *E. sinensis* was significantly increased after *S. eriocheiris* infection. In this study, myosin and tubulin cytoskeleton proteins were significantly upregulated, which was hypothesized to promote cell adhesion and regulate hemolymph phagocytosis during *M. bicuspidata* infection.

GO analysis showed that many immune pathways were enriched in biological processes, such as regulation of the toll signaling pathway, cell surface receptor signaling pathway, response to fungus, negative regulation of hydrolase activity, and integrin-mediated signaling pathway. Many serine protease and serine protease inhibitors (serpins) are involved in these immune pathways, including clip domain-containing serine protease (CSP), leukocyte elastase inhibitor (Serpin B1) and Serpin 42Dd. CSP plays an important role in activating phenoloxidase (PO) system and induces melanization of immune response in crustaceans (35, 36). In addition to activating PO, CSP can also cleave the precursor of spätzle to activate the spätzle, which is required for Toll signaling pathway and antimicrobial peptides synthesis (37, 38). In this study, CSP and spätzle 5-like protein were significantly increased, which indicated that *E. sinensis* could activate PO system and improve antimicrobial peptides synthesis in resistance of *M. bicuspidata* infection. Serpins have been found in all higher eukaryotes, bacteria, and viruses, and they play an important role in the immune response by regulating the protein hydrolysis cascade (39, 40). Serpin B1 is an intracellular protein that acts primarily to protect the cell from proteases released into the cytoplasm during stress (41). Serpin 42Dd isoforms may be essential for immune defense by inhibiting a large spectrum of pathogenic proteolytic enzymes (42). In this study, the expression levels of Serpin B1 and Serpin 42Dd were 5.0 and 4.1 times higher than those in the control group, respectively, indicating that Serpin B1 and Serpin 42Dd play an essential role in the immune defense against *M. bicuspidata* in *E. sinensis*, possibly by inhibiting the protease produced by *M. bicuspidata* and/or protecting cells from excess protease effects.

Among the 38 upregulated proteins, the expression levels of many immune genes (transferrin, heat shock cognate 70 kDa protein, and catalase) were significantly increased. Transferrin exists widely in most organisms, from invertebrates to vertebrates. It is a type of glycoprotein with a molecular weight of 70–80 kDa. It plays an important role in maintaining iron homeostasis and is an essential growth and non-specific immune factor (43). Toe et al. found that transferrin plays a vital role in the innate immunity of crustaceans by stimulating *M. rosenbergii* with *Aeromonas hydrophila* (44). Xu et al. used protein technology to screen the immune proteins of *E. sinensis* hemocytes stimulated by *S. eriocheiris* (45). The results showed that the expression level of transferrin was significantly increased after *S. eriocheiris* stimulation. In this study, the expression of transferrin protein in *E. sinensis* infected with *M. bicuspidata* was 3.0 times higher than that in the control group, indicating that it also plays an important role in the innate immunity of *E. sinensis* against infection by *M. bicuspidata*.

Catalase (CAT) is an important part of the body’s antioxidant defense system. After superoxide dismutase converts oxygen free radicals into H_2_O_2_, CAT further reduces them into water and oxygen molecules, which can protect cells from hydrogen peroxide poisoning and protect the stability of the internal environment of the body (46). Therefore, changes in CAT gene expression and activity can reflect the metabolism of free radicals in the body and thereby indicate the health status of organisms. It is also an important index of antioxidant defense ability (47). After infection by *M. bicuspidata*, the CAT gene expression level in the hemolymph tissue of *E. sinensis* increased to 2.4 times higher than that of the control group. The results showed that *M. bicuspidata* infection causes phagocytosis and consumes excessive reactive oxygen species in the body to maintain the stability of the intracellular environment and then cause respiratory burst. The high amount of hydrogen peroxide protein is mainly used to scavenge oxygen free radicals to avoid body damage.

Previous studies have shown that environmental stress (such as heat stress, heavy metals, and ammonia nitrogen) can promote the synthesis of the heat shock protein HSP70 (48, 49). HSP70 can effectively reduce damage to the body caused by stress by preventing protein folding and degrading denatured protein. Recent studies have shown that HSP70 also plays a key role in immune regulation, including as a stimulator and target to stimulate innate and adaptive immunity (50, 51). Among crustaceans, HSP70 levels significantly increased in *Litopenaeus vannamei* that had been exposed to hypodermic and thermopoietic necrosis virus, WSSV, and pathogen infection, indicating that it plays an important role in the innate immunity of shrimp (52). In this experiment, the expression of HSP70 protein in the *E. sinensis* infected by *M. bicuspidata* was twice as high as that in the control group, indicating that HSP70 is also involved in the innate immune response to yeast infection.

There was no significant KEGG pathway for the 24 significantly decreased proteins. In the GO enrichment analysis, these downregulated proteins were mainly concentrated in the biological processes related to organ genesis and formation, such as PDGF/VEGF-related factor protein and the integrin-linked protein kinase homolog pat-4 gene. The results showed that host cell regeneration was affected after yeast infection (53, 54). The typical feature of “milky disease” is that the hemolymph does not solidify and presents as a milky liquid. Crustaceans have an open circulatory system; they must heal wounds through effective coagulation reactions to prevent loss of hemolymph and capture pathogenic microorganisms to prevent them from spreading into the hemolymph. The coagulation reaction is initiated by the release of Ca^2+^-dependent transglutaminase by hemocytes, which causes clotting protein polymerization in the hemolymph to form a stable clot (55, 56). Our research found that after 48 h of *M. bicuspidata* infection, the content of coagulation protein in the infected group decreased significantly, which was 0.28 times that of the control group, and the transglutaminase also decreased significantly. This showed that *M. bicuspidata* could reduce the coagulation reaction of the host, which is helpful for the proliferation of yeast.

## Conclusions

This study investigated the protein expression changes in *E. sinensis* infected with *M. bicuspidata* using proteomics technology. The results showed that many immune-related proteins in *E. sinensis* were significantly upregulated after infection with *M. bicuspidata*, such as cytoskeleton-related proteins, serine protease and serine protease inhibitor proteins. The upregulation of these proteins indicated that the phenoloxidase system, phagocytosis and the ROS systems were induced by *M. bicuspidata*. In addition, proteins related to organ and tissue regeneration and coagulation reaction proteins were significantly downregulated by *M. bicuspidata* infection, which inhibited hemocyte regeneration and hemolymph agglutination. This finding provides an insight into the immune defense mechanism of crustacean hemolymph against pathogenic yeast.

## Data Availability Statement

The raw data supporting the conclusions of this article will be made available by the authors, without undue reservation.

## Ethics Statement

The animal study was reviewed and approved by the Animal Experiments Ethics Committee of Shenyang Agricultural University.

## Author Contributions

HJ, JB, and QC were involved in designing of the research and wrote the manuscript. HJ, JB, YX, CF, and XL performed the majority of the experiment, data processing, analysis, and interpretation. All authors contributed to the article and approved the submitted version.

## Funding

This work was supported by Modern Agro-industry Technology Research System (CARS-48); Liaoning province Department of Education fund item (LSNQN202002); Liaoning Science and Technology Mission Project (2020JH5/10400147; 2020JH5/10400115); and Liaoning Province Key R&D Planning Guidance Plan Project (2019JH8/10200018).

## Conflict of Interest

The authors declare that the research was conducted in the absence of any commercial or financial relationships that could be construed as a potential conflict of interest.
